# Kansas City Veterinary College

**Published:** 1895-04

**Authors:** 


### KANSAS CITY VETERINARY COLLEGE.

The Kansas City Veterinary College held its fourth Annual Commencement exercises at the Jackson County Medical Hall, Masonic Building, Thursday evening, March 21, 1895.

The following gentlemen received their diplomas :

Wm. L. Elliott, Spring Hill, Kansas ; Albert B. Wilmoth, Turney, Missouri ; Scott W. Peck, Las Animas, Colorado ; George M. Needham, Rockford, Illinois ; John E. Topping, Paola, Kansas ; Harry V. Goode, Lexington, Missouri ; Clarence A. Atwood, Pecatonica, Illinois ; John W. Stalker, Adrian, Kansas ; William T. King, Clinton, Missouri ; Henry R. Thompson, Aurora, Kansas ; William A. Mark, Elgin, Illinois ; Silas L. Brooking, M.D., Eureka, Kansas ; James H. Cock, Clinton, Missouri ; John O. Young, Rice, Kansas ; Benjamin F. Kaup, Nevada, Missouri.

The exercises were interspersed with instrumental music by Des Gosses' Orchestra.

After prayer by Rev. W. A. Quayle, the annual address was delivered by Hon. Webster Davis, Mayor.

The salutatory was delivered by John E. Topping, of the graduating class. The valedictory by Wm. L. Elliott.

The ceremonies were directed by Sisco Stewart, M.D., D.V.M., of the college faculty.

---

